# Limb Remote Ischemic Postconditioning Reduces Ischemia-Reperfusion Injury by Inhibiting NADPH Oxidase Activation and MyD88-TRAF6-P38MAP-Kinase Pathway of Neutrophils

**DOI:** 10.3390/ijms17121971

**Published:** 2016-11-25

**Authors:** Gangling Chen, Xinyi Ye, Jiangwei Zhang, Tingli Tang, Lin Li, Peirong Lu, Qi Wu, Boyang Yu, Junping Kou

**Affiliations:** 1 Department of Pharmacology of Chinese Materia Medica, China Pharmaceutical University, 639 Longmian Road, Nanjing 211198, China; chengangling@cpu.edu.cn (G.C.); 15651648635@163.com (T.T.); 15850670086@163.com (L.L.); lupeirong321@163.com (P.L.); 2 Jiangsu Key Laboratory of TCM Evaluation and Translational Research, Department of Complex Prescription of TCM, China Pharmaceutical University, 639 Longmian Road, Nanjing 211198, China; yexinyi1991@126.com (X.Y.); 15651671978@163.com (J.Z.); 3 State Key Laboratory of Natural Medicines, Research Department of Pharmacognosy, China Pharmaceutical University, 639 Longmian Road, Nanjing 211198, China; christina_576636@126.com

**Keywords:** ischemic stroke, reperfusion injury, limb, neutrophil, NADPH oxidase, MyD88/TRAF6/p38-MAPK pathway

## Abstract

Limb remote ischemic postconditioning (LRIP) has been confirmed to reduce the ischemia-reperfusion injury but its mechanisms are still not clear. This study clarified the mechanism of LRIP based on the nicotinamide-adenine dinucleotide phosphate (NADPH) oxidase and Myeloid differentiation factor 88 (MyD88)-Tumor necrosis factor (TNF) receptor-associated factor 6 (TRAF6)-P38 pathway of neutrophils. Rat middle cerebral artery occlusion (MCAO) model was used in this study. Ischemia-reperfusion injury was carried out by MCAO 1.5 h followed by 24 h reperfusion. LRIP operation was performed to the left femoral artery at 0, 1 or 3 h after reperfusion. Behavioral testing, including postural reflex test, vibrissae-elicited forelimb placing test and tail hang test, showed that LRIP operated at 0 h of reperfusion could significantly ameliorate these behavioral scores. Pathological examinations, infarct size, Myeloperoxidase (MPO) activity showed that LRIP operated at 0 h of reperfusion could significantly ameliorate the pathological scores, reduce the infarct size and MPO activity in the brain and increase the MPO activity in the left leg. By using Neutrophil counting, immunofluorescence and real-time PCR techniques, we found that LRIP operated at 0 h of reperfusion could reduce neutrophil counts in the peripheral blood and downregulate the activation of neutrophil in the peripheral blood and rat brain. Western blots revealed that MyD88, TRAF6, p38 mitogen-activated protein kinase (p38-MAPK) in neutrophils and the phosphorylation of p47phox (Ser 304 and Ser 345) in neutrophil could be downregulated by LRIP. Our study suggests that LRIP inhibits the number and activation of neutrophils in the rat brain and peripheral blood linked to down-regulating the activation of NADPH oxidase in neutrophils by MyD88/TRAF6/p38-MAPK pathway.

## 1. Introduction

Stroke, which includes ischemic stroke and hemorrhagic stroke, is one of the fatal or disabling cerebral vascular diseases [1,2]. Ischemic stroke accounts for over 80% of the total incidences of stroke. Thrombolysis and interventional therapy can treat ischemic stroke by recanalization of the embolic cerebral blood vessels [3,4]. However, this kind of treatment is accompanied by ischemia-reperfusion injury (IRI). Limb remote ischemic postconditioning (LRIP) is a developed postconditioning procedure [5]. The transient ischemia-reperfusion (I/R) operation applied to non-vital remote tissues (for example, the limb), immediately after reperfusion of a vital organ, can safely reduce the IRI of a vital organ (for example, the brain) through remote postconditioning. LRIP has achieved significant therapeutic effects in clinical and experimental studies [6,7,8,9]. For its easy operation and obvious curative effects, researchers have paid much attention recently to this method of treatment [10,11]. Furthermore, LRIP is able to reduce reperfusion injury by reducing oxidative damage [12], attenuating neuronal apoptosis, suppressing p38 mitogen-activated protein kinase (p38 MAPK)-Activating transcription factor 2 (ATF2) pathway [13], activating the NF-E2-related factor 2 (Nrf2)-ARE (antioxidant response element) pathway [14], down-regulating aquaporin 4 (AQP4) astrocytes [15] and suppressing Hypoxia-inducible Factor 1α (HIF-1α) [16], among others. However, its potential mechanism to relieve brain I/R remains unclear.

The mechanisms of IRI include oxidant production, metabolic acidosis, leucocyte–endothelial cell adhesion, increase in microvascular permeability and no-reflow phenomena, among others [17,18,19,20]. Among them, oxidant production and neutrophils have caught the attention of researchers [21,22]. Abundant pieces of evidences have shown that under IRI, neutrophils will be activated and a great deal of reactive oxidative species (ROS) will be produced [23,24]. Many researches have proven that nicotinamide-adenine dinucleotide phosphate (NADPH) oxidase [25,26,27,28], especially the NADPH oxidase of neutrophils is the main source of ROS under an IRI circumstance [29,30,31]. This kind of oxidative damage will affect the normal function of proteins and cells. It is noteworthy that LRIP is also able to inhibit the activity of NADPH oxidase, reduce the activation of neutrophils [32,33,34] and reduce the accumulation of neutrophils in the area at risk [35].

In summary, oxidative damage is one of the causes of reperfusion injury. Neutrophils and NADPH oxidase, especially the NADPH oxidase in neutrophils is critical for the production of ROS. On the other hand, many researchers have concerns about LRIP, but its mechanisms to relieve brain IRI are unclear. In this study, we presume that LRIP is able to reduce the activity of neutrophil NADPH oxidase and therefore, exert protective effects to IRI. The study is aimed at better understanding of the mechanisms of LRIP.

## 2. Results

### 2.1. Effect of Limb Remote Ischemic Postconditioning **(**LRIP) on the Neurobehavioral Deficits in Middle Cerebral Artery Occlusion (MCAO) Rats

**Figure 1:**
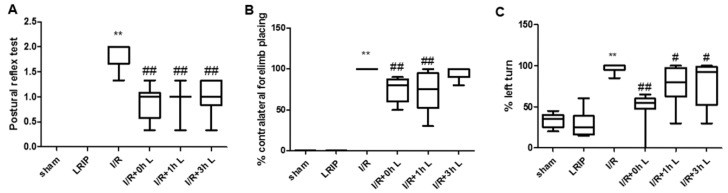
Effects of limb remote ischemic postconditioning **(**LRIP) on neurobehavioral testing scores assessed at 24 h after reperfusion. LRIP operation was carried out by three cycles of 5 min occlusion/5 min release of the left femoral artery at 0, 1 or 3 h after reperfusion, respectively: (**A**) postural reflex test; (**B**) vibrissae-elicited forelimb placing test; and (**C**) tail hang test. Data are expressed as mean ± SD, *n* = 6, ** *p* < 0.01 vs. sham group, ^##^ *p* < 0.01 vs. Ischemia-reperfusion (I/R) group, ^#^ *p* < 0.05 vs. I/R group.

The effects of LRIP treatment at 0, 1 or 3 h after reperfusion were ascertained by postural reflex test, vibrissae-elicited forelimb placing test and tail hang test. There was no significant difference between the LRIP group and the Sham group, while the IRI neurobehavioral scores were significantly higher (*p* < 0.01; Figure 1). Moreover, LRIP treatment at 0, 1 or 3 h after reperfusion could attenuate neurobehavioral scores to various degrees. Compared with the I/R group, LRIP treatment at 0 h could significantly reduce the scores of the three kinds of behavioral testing (*p* < 0.01; Figure 1) and LRIP treatment at 1 h had significant effects in the postural reflex test, the vibrissae-elicited forelimb placing test (*p* < 0.01; Figure 1B) and a relatively minor effect in the tail hang test (*p* < 0.05; Figure 1C). Finally, LRIP treatment at 3 h had a significant effect only in the postural reflex test (*p* < 0.05; Figure 1A).

### 2.2. Effect of LRIP on Neutrophil Infiltration in the Brain and Left Hind Limb Gastrocnemius Muscle in MCAO Rats

**Figure 2:**
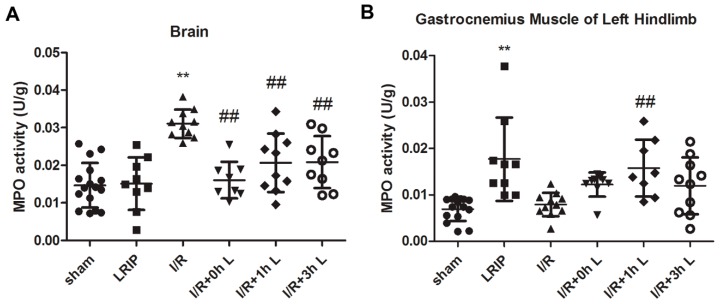
Effects of LRIP on neutrophil infiltration in the brain and left hindlimb gastrocnemius muscle: Myeloperoxidase (MPO) activities in: the brain (**A**); and left hindlimb gastrocnemius muscle (**B**) were assessed at 24 h after reperfusion. Data are expressed as mean ± SD, *n* = 6, ** *p* < 0.01 vs. sham group, ^##^ *p* < 0.01 vs. I/R group.

The effects of LRIP given at different time points were further evaluated by neutrophil infiltration in the brain and left hindlimb gastrocnemius muscle. The brain Myeloperoxidase (MPO) activity of the I/R group was significantly higher than the Sham and LRIP groups (*p* < 0.01, Figure 2A). LRIP treatment, especially at 0 h after reperfusion, has a significantly lower level of MPO activity in the brain than that in the I/R group (*p* < 0.01), suggesting that neutrophils severely infiltrated the brain after I/R, which was attenuated by LRIP treatment at 0 h after reperfusion. Though the value of LRIP group is relatively higher, which may be due to the short term I/R of the left femoral artery, there was no significant difference among MPO activities in the hindlimb gastrocnemius muscle of the Sham, LRIP and I/R groups (Figure 2B). Interestingly, LRIP treatment at 0 h after reperfusion resulted in a significantly higher MPO activity in the left hindlimb gastrocnemius muscle than the I/R group. These data indicate that some neutrophils have adherence with the left femoral artery or infiltrate the gastrocnemius muscle because LRIP reduced neutrophil infiltration in the brain.

### 2.3. Effects of LRIP on the Histopathological Changes and Infarct Volume of the Brain in MCAO Rats

**Figure 3:**
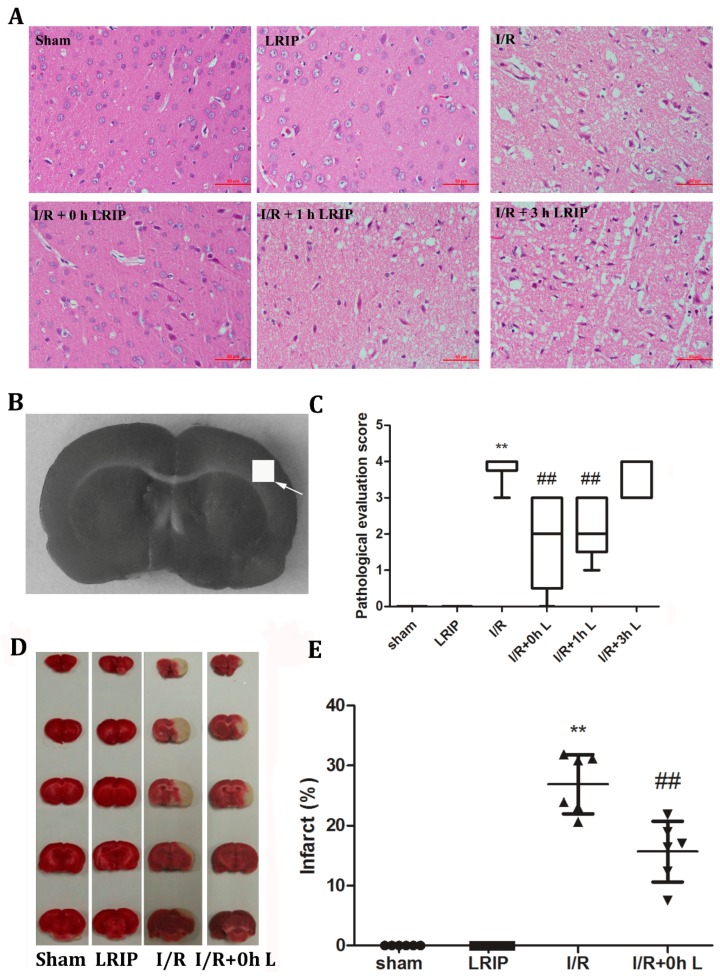
LRIP ameliorated histopathological changes and infarct size of the brain. (**A**) Brain sections of rats in different groups were stained by Hematoxylin and Eosin and then examined using a light microscope. Representative photomicrographs of ischemic cortices from the sham, LRIP, I/R, I/R + 0 h, I/R + 1 h, and I/R + 3 h groups were established. The most significant reversal of injury was observed in the I/R + 0 h group. The magnification was 400×. Scale bar = 50 µm; *n* = 6; (**B**) rat brain slices were obtained by making a coronal cut through the optic chiasma. Tissue corresponding to the square icon was used for Hematoxylin and Eosin Staining; (**C**) histopathological scores of paraffin sections of the rat brain; (**D**) representative infarcts stained by 2,3,5-triphenyhetrazolium chloride (TTC); (**E**) bar graphs show the average infarct size of the five slices. Data are expressed as mean ± SD, *n* = 6, ** *p* < 0.01 vs. sham group, ^##^ *p* < 0.01 vs. I/R group.

As shown in Figure 3A, brain sections from the I/R group exhibited neuronal loss, numerous vacuolated spaces, chromatin condensation and nucleic fragmentation while the LRIP groups showed a different degree of amelioration. I/R + 0 h LRIP exerted the most significant protective effect. These results indicate that LRIP operated at an early phase of reperfusion would be more beneficial to the I/R group. Thus, we chose 0 h after reperfusion to carry out LRIP treatment to further explore the mechanism involved. Figure 3D,E shows that LRIP operated at 0 h of reperfusion could significantly reduce the infarct volume compared with the I/R group.

### 2.4. Effect of LRIP on the Infiltration and the Expression of Nicotinamide-Adenine Dinucleotide Phosphate (NADPH) Oxidase p47^phox^ of Neutrophils in MCAO Rats

**Figure 4:**
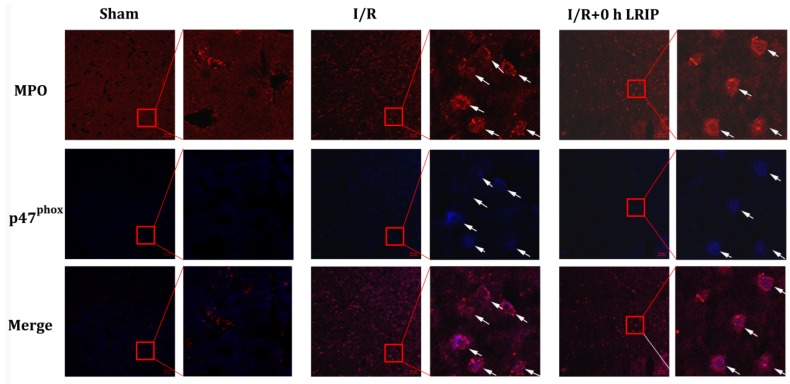
LRIP reduced the infiltration and p47phox membrane translocation of neutrophil. Representative immunofluorescence microscopeimages of p47^phox^ (blue) in neutrophils with the marker MPO (red). Scale bar = 50 μm in 200× images and 20 μm in 600× images. Infiltration of neutrophils was increased in the I/R group and the expression and membrane translocation of its p47^phox^ were augmented. These phenomena were attenuated in the I/R + 0 h LRIP group.

The amount and activation of infiltrated neutrophils in the brain were assessed by immunostaining with antibodies of NADPH oxidase p47^phox^ or neutrophil (Figure 4), which shows that infiltrated neutrophils and the expression of p47^phox^ in neutrophil were increased in the I/R group. LRIP treatment inhibited neutrophil infiltration and reduced the expression of p47^phox^ after I/R.

### 2.5. Effect of LRIP on the Number of Neutrophil and NADPH Oxidase Activation from the Peripheral Blood in MCAO Rats

**Figure 5:**
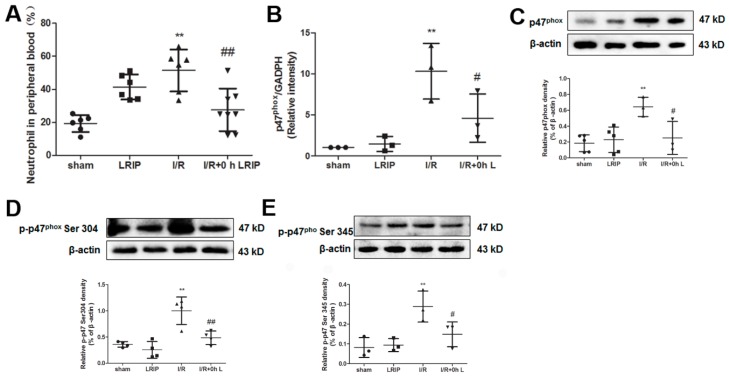
Effect of LRIP on the number of neutrophil and nicotinamide-adenine dinucleotide phosphate (NADPH) oxidase activation of peripheral blood: (**A**) Neutrophil count in the peripheral blood. Data are expressed as percentage of neutrophil in the peripheral blood (*n* = 6); (**B**) qPCR result showing the mRNA expression change of p47^phox^ in the Sham, LRIP, I/R, and I/R + 0 h LRIP groups. Glyceraldehyde-3-phosphate dehydrogenase (GAPDH) is used as the internal control (*n* = 3); (**C**–**E**) Western blot assessment of p47^phox^ and its phosphorylationat Ser 304 and Ser 345 in neutrophils from the peripheral blood were performed after 24 h reperfusion (*n* = 3). Data are expressed as mean ± SD, ** *p* < 0.01 vs. sham group, ^#^ *p* < 0.05 vs. I/R group, ^##^ *p* < 0.01 vs. I/R group.

Peripheral blood was collected after 24 h reperfusion. The number of neutrophils in the peripheral blood was elevated after LRIP treatment compared with the Sham group. Particularly, the number of neutrophils in the I/R group was significantly higher than that in the Sham group. LRIP treatment could reverse the increase of neutrophils caused by I/R (Figure 5A). The mRNA and protein expression of p47^phox^ in Ser 304 and Ser 345-phosphorylated p47^phox^ were increased in the neutrophils of the I/R group, suggesting the augmentation of neutrophil NADPH oxidase activation. Furthermore, LRIP could down-regulated the expression of mRNA and protein of p47^phox^ and also inhibit the phosphorylation of p47^phox^ (Figure 5B–E).

### 2.6. Effect of LRIP on Myeloid Differentiation Factor 88 (MyD88)-Tumor Necrosis Factor (TNF) Receptor-Associated Factor 6 (TRAF6)-p38 Mitogen-Activated Protein Kinase (p38-MAPK) Signaling Pathways in MCAO Rats

**Figure 6:**
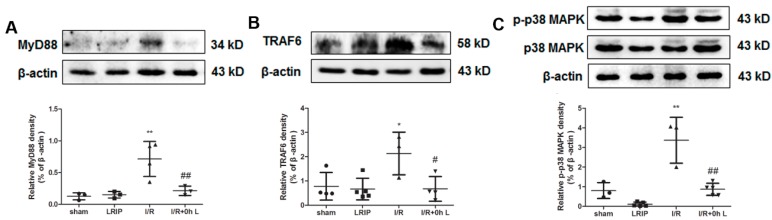
The Myeloid differentiation factor 88 (MyD88)/Tumor necrosis factor (TNF) receptor-associated factor 6 (TRAF6)/p38 mitogen-activated protein kinase (p38-MAPK) signaling pathway is involved in the mechanism through with LRIP protects against I/R injury. Representative images of Western blot assessments and the quantitative analysis of the ratio of: MyD88 (**A**); TRAF6 (**B**); and p-p38 MAPK (**C**). Data are expressed as mean ± SD, *n* = 3, * *p* < 0.05 vs. sham group, ** *p* < 0.01 vs. sham group, ^#^ *p* < 0.05 vs. I/R group, ^##^ *p* < 0.01 vs. I/R group.

To investigate the mechanism of the protection of LRIP from I/R injury, we detected the expressions of Myeloid differentiation factor 88 (MyD88), Tumor necrosis factor (TNF) receptor-associated factor 6 (TRAF6) and p38 mitogen-activated protein kinase (p38-MAPK) in neutrophils from the peripheral blood. As shown in Figure 6, compared with the Sham and LRIP groups, the expression of MyD88, TRAF6 and p-p38 MAPK were up-regulated after I/R and significantly decreased after LRIP treatment. The results suggest that MyD88/TRAF6/p38 MAPK signaling pathway is involved in the mechanism of the protective effect of LRIP against I/R injury.

## 3. Discussion

LRIP has achieved significant therapeutic effects on IRI during experimental and clinical studies. LRIP is easy for operation, while its mechanisms are unclear [36,37]. The role of inflammation and oxidative damage on stroke and cardiovascular disease pathogenesis is very important and has been studied by lots of researchers [20,21,38,39]. An abundant body of research has shown that when some organs endures IRI, the NADPH oxidase of neutrophils will be activated and then a great deal of superoxide anion will be produced. In this study, it is assumed that LRIP might reduce the IRI of the rat brain by inhibiting the activation of NADPH oxidase in neutrophils, so as to reduce oxidative damage.

First, we compared the protective effects of LRIP operated at 0, 1 or 3 h of reperfusion. Based on the results of behavioral testing, pathological score and infarct volume of the brain, the protective effect of LRIP operated at 0 h of reperfusion was better than operation at 1 or 3 h (Figure 1 and Figure 3), which are similar to those of other studies [10,40]. Second, the mechanisms of LRIP were studied based on the number and activation of neutrophils. The MPO activity assay showed that when the rat brain endured IRI, MPO activity would be increased (Figure 2A) and LRIP operation to the left leg could reduce the MPO activity in the rat brain, which is consistent with previous reports [41,42,43]. On the other hand, other studies have proven that LRIP could reduce MPO activity in the gastric tissue that had an IRI and rapid postconditioning could also reduce MPO activity in the ischemic cortex [44,45]. These findings are similar to our results. Our study also showed that MPO activity in the left leg increased (Figure 2B). MPO activity is commonly used to reflect the number of neutrophils [46,47,48]. It could be inferred that LRIP can “attract” neutrophils from the brain to the hind legs by some means, so as to protect the more important organ: the brain. The neutrophil count of the peripheral blood showed that the numbers of neutrophils is increased after I/R, which might be due to the neutrophils that were released from the marginated pool [49]. Under physiological conditions, a high number of neutrophils are stored in the marginated pool, and IRI could induce the neutrophil release from the marginated pools to the circulating pools, so the numbers of neutrophils in the peripheral blood is increased. LRIP could suppress the increase of neutrophils under IRI (Figure 5A). Based on these results, it could be concluded that LRIP is able to reduce the release of the peripheral neutrophils by means of anti-inflammatory or anti-oxidant. Furthermore, because the half-life of circulating neutrophil is just 6–8 h [50], LRIP could also probably affect the release of mature neutrophils from the bone marrow. Third, the activation of neutrophils in the rat brain and the peripheral blood was studied. Results showed that the activation of neutrophils increased in the rat brain that has suffered from an IRI, which could be down-regulated by LRIP (Figure 4). Then we used Western blotting to analyze the activation of neutrophils in the peripheral blood. The activation of neutrophils was increased with IRI and decreased after LRIP (Figure 5B–E).

Finally, the activation of neutrophil NADPH oxidase and its mechanisms were studied. Some researchers have proven that the activation of neutrophil is based on the Toll-like receptor 8 (TLR8)/p38 MAPK/Extracellular signal-regulated kinase 1/2 (ERK1/2)/NOX2 signaling pathway and MyD88-IL-1 receptor (IL-1R)-associated kinase4 (IRAK4)-p38 MAPK and MyD88-IRAK4-Akt signaling pathways [29,51]. In this study, we chose MyD88/TRAF6/p38 MAPK signaling pathway as the target pathway. Results showed that p47^phox^ in neutrophils could be phosphorylated by MyD88/TRAF6/p38 MAPK signaling pathway with IRI. LRIP could inhibited the activation of neutrophil NADPH oxidase by MyD88/TRAF6/p38 MAPK signaling pathway (Figure 6). It could be concluded that when the rat brain has suffered from an IRI, the MyD88/TRAF6/p38 MAPK signaling pathway of neutrophils would be activated, then the p47^phox^ would be phosphorylated and then NADPH oxidase will release a high number of superoxide anion causing oxidative damage. LRIP could protect the rat brain by down-regulation of the above pathway.

In conclusion, these findings have clarified the protective mechanisms of LRIP through the NADPH oxidase pathway of neutrophils. However, there are still several unresolved issues, such as the reason LRIP reduced the number of neutrophils in the peripheral blood of rat that has suffered from an IRI, the effects of LRIP to the MyD88/TRAF6/p38 MAPK signaling pathway of neutrophils in the brain and the effects of plasma or serum of LRIP rats to control neutrophils. We will resolve these problems in future research.

## 4. Materials and Methods

### 4.1. Animals

Male Sprague-Dawley rats weighing 250–280 g were provided by the Qinglongshan Experimental Animal Breeding Farm (Nanjing, China). All rats were housed in the Laboratory of Experimental Animal of China Pharmaceutical University. All animal experiments were approved by the Animal Ethics Committee of China Pharmaceutical University. All animal experiments were conducted under a protocol approved by Animal Ethics Committee of China Pharmaceutical University and conformed to internationally accept ethical standards.

### 4.2. Transient Focal Ischemia and Postconditioning in Rats

To induce transient focal ischemia, rats were anesthetized with chloral hydrate (330 mg/kg, intraperitoneal injection) and were subjected to middle cerebral artery occlusion (MCAO) as described previously [52]. In brief, Sprague-Dawley rats were randomly divided into the following groups: Sham group, LRIP group, ischemia-reperfusion (I/R) group, I/R + 0 h LRIP group, I/R + 1 h LRIP group, and I/R + 3 h LRIP group. For the LRIP group, LRIP operation was carried out by three cycles of 5 min occlusion/5 min release of the left femoral artery using clamps [9,10]. For the I/R group, middle cerebral artery ischemia was induced for 1.5 h, followed by reperfusion for 24 h. For the I/R + 0 h LRIP group, the I/R + 1 h LRIP group, and the I/R + 3 h LRIP group, rats were treated with LRIP operation at 0, 1 and 3 h after reperfusion respectively. The sham group just exposed the right common carotid artery.

### 4.3. Behavioral Testing

The behavioral tests were carried out 24 h after reperfusion in a double-blind fashion using the postural reflex test [53], vibrissae-elicited forelimb placing test [54] and tail hang test [55].

### 4.4. Determination of Myeloperoxidase (MPO) Activity

Myeloperoxidase (MPO) activity has been used as an index of neutrophil infiltration in or adhesion with tissues and organs [56]. In this study, we used MPO activity to indicate neutrophils infiltration in the brain and the left hindlimb gastrocnemius muscles. Rats were anesthetized with chloral hydrate and transcardially perfused with phosphate-buffered saline (PBS, pH 7.4). MPO activity was measured according to the manufacturer's instructions of (Jian Cheng Bioengineering Institute, Nanjing, China). Briefly, the brain ischemic cortical tissues and left hindlimb gastrocnemius muscle were collected and adequate solution from the kit was added to obtain 5% (*w/v*) homogenates. After centrifugation at 3000 rpm at 4 °C for 15 min, the supernatant was added into PBS (pH 6.0) containing 0.0005% H_2_O_2_ and 0.17 mg/mL 3,3′-dimethoxybenzidine. MPO activity was determined by measuring the H_2_O_2_-dependent oxidation of 3,3′-dimethoxybenzidine and expressed as units per gram weight of wet tissue.

### 4.5. Hematoxylin and Eosin Staining and Infarct Size Measurement

Histological changes of the rat brain were assessed by hematoxylin and eosin (HE) staining. Rats were anesthetized with chloral hydrate at 24 h after reperfusion and transcardially perfused with PBS. Brains were immediately taken from rats and after fixation by immersing in 4% paraformaldehyde for 24 h and then processed routinely for paraffin wax embedding. A series of adjacent brain sections (3 µm thick) were cut from the coronal plane of the wax-embedded tissue and were stained with HE for analysis of histological changes conducted by a pathologist blinded to the treatment groups [57].

The degree of brain damage in the cerebral cortex was scored on a 5-point scale as follows: 0 = normal, 1 = few neurons damaged (lesion area/area of cerebral hemisphere < 1/3), 2 = several neurons damaged (1/3 < lesion area/area of cerebral hemisphere < 1/2), 3 = moderate neurons damaged (1/2 < lesion area/area of cerebral hemisphere < 2/3), 4 = majority of neurons damaged (2/3 < lesion area/area of cerebral hemisphere). For the infarct size measurement, the rat brains were sectioned coronally at 2 mm intervals and incubated with 2% 2,3,5-triphenyhetrazolium chloride (TTC) for 20 min, and then the infarct was calculated as defined previously [9].

### 4.6. Immunofluorescence Staining

Rats were anesthetized and transcardially perfused with PBS, followed by 0.1 M PBS containing 4% PFA (pH = 7.4). Perfusion-fixed brains were postfixed in paraformaldehyde overnight, followed by dehydration in 40% sucrose. Coronal brain sections (10 µm thick) were cut on a cryostat (CM1950; Leica Microsystems, Wetzlar, Germany), and the sections were blocked for 2 h in 5% bovine serum albumin in PBS with 0.3% Triton X-100. The sections were then incubated overnight at 4 °C in 3% bovine serum albumin in 0.1% Triton X-100/PBS with the following primary antibodies: anti-p47^phox^ antibody (1:200; sc-14015)and anti-MPO antibody (1:100; sc-34159) (both were purchased from Santa Cruz Biotechnology, Santa Cruz, CA, USA). After being rinsed three times with PBS, sections were incubated for 2 h in fluorochrome conjugated secondary antibody. After being rinsed with PBS, the sections were examined under a laser scanning confocal microscope (Carl Zeiss LSM 700, Oberkochen, Germany).

### 4.7. Peripheral Blood Count

Rats were anesthetized at 24 h after reperfusion. Blood was collected from femoral arteries and anticoagulanted with heparin sodium, and then blood cells were counted by a Blood Analyzer (ADVIA2120, Bayer, Tarrytown, NY, USA).

### 4.8. Neutrophil Isolation

Rats were anesthetized at 24 h after reperfusion. Blood was collected from femoral arteries and anticoagulated with heparin sodium. Neutrophils were isolated using neutrophil extraction kit (Sangon Biotech Co., Ltd., Shanghai, China) according to the manufacturer’s instruction. The neutrophil purity was qualified by Wright-Giemsa stain method, the purity of neutrophils >90%.

### 4.9. Real-Time PCR

Total RNA was extracted from isolated neutrophils using TRIzol (Invitrogen, Carlsbad, CA, USA) according to the manufacturer’s instruction. The RNA quantity and quality were detected using Nano-100 (AllSheng Instuments, Hangzhou, China). The synthesis of first-strand DNA was performed by EasyScript First-Strand cDNA Synthesis SuperMix kit (Sangon Biotech Co., Ltd., Shanghai, China) in a 20-μL reaction mixture. Then, 2 μL of the product was used for real-time PCR (qPCR) in a final volume of 10 μL using the gene-specific primers for p47^phox^, MyD88 and GAPDH. The primers used for amplification of the rat genes were as follows: p47^phox^, forward 5′-ATTTTCTTGGTGATTGATG-3′ and reverse 5′-GAGGGATGTTACTTACTGG-3′; GAPDH, forward 5′-TCCTGCACCACCAACTGCTTAG-3′ and reverse 5′-AGTGGCAGTGATGGCATGGACT-3′. The amplification conditions were as follows: 94 °C, 30 s, 1 cycle; and 94 °C, 5 s and 60 °C, 30 s for 40 cycles. The melting curve was then determined. Gene transcripts were quantified with TranStart Top Green qPCR SuperMix kit (TransBionovo Co., Ltd., Beijing, China). Data were calculated using the 2^−ΔΔ*C*t^ method and presented as fold change of transcripts for the p47^phox^ gene in the peripheral blood of other groups compared with rats of the Sham group (defined as 1.0-fold). Rat GAPDH was used as an internal control. The relative expression of the target gene was normalized to the level of GAPDH in the same cDNA preparation.

### 4.10. Western Blotting

Proteins extracted from isolated neutrophils were used for Western blot analysis. Proteins were separated by 12.5% SDS/PAGE electrophoresis and transferred to PVDF membranes, then the membranes were blocked with 5% BSA at room temperature for 2 h and then incubated overnight at 4 °C with the corresponding primary antibodies: p47^phox^ (1:800; sc-14015, Santa Cruz Biotechnology), p-p47^phox^Ser 304 (1:500, bs-1047R, BioSS, Woburn, MA, USA), p-p47^phox^Ser 345 (1:1000, 118391, Sigma-Aldrich, St. Louis, MO, USA), TRAF6 (1:1000, ab40675, Abcam, Cambridge, UK), MyD88(1:500, bs-1047R, Bioss Biotech, Woburn, MA, USA), p38 MAPK (1:1000, AM065, Beyotime Institute of Biotechnology, Beijing, China), p-p38 MAPK (1:1000, AM063, Beyotime Institute of Biotechnology, Beijing, China) and β-actin (1:5000, AP0060, Bioworld, Atlanta, GA, USA). After three washes with TBST, the membranes were incubated with horseradish-peroxidase-conjugated secondary antibody for 2 h. Immunoreactive bands were detected by a chemiluminescence system (ECL Plus; Amersham, Arlington Heights, IL, USA) and analyzed by Quantity One analysis software (Bio-Rad Laboratories Inc., Hercules, CA, USA). β-Actin was used as the internal control.

### 4.11. Statistical Analysis

Data are expressed as mean ± SD. Statistical analysis was performed using GraphPad Prism software Version 5.01 (GraphPad Software, Inc., La Jolla, CA, USA). The differences in the effects between each group were assessed using one-way analysis of variance (ANOVA) followed by Dunnett’s test. The significance level was set at *p* < 0.05.
